# Response of Leucocyte Populations in the Ileal Peyer's Patch of Fetal Lambs Treated with Ferritin *Per* Os

**DOI:** 10.1155/1995/96934

**Published:** 1995

**Authors:** Lena H. M. Renström, Charles Mcl. Press, Wendy Trevella, Thor Landsverk

**Affiliations:** 1 Department of Morphology Genetics and Aquatic Biology Norwegian College of Veterinary Medicine Dep. 0033 Oslo P.O. Box 8146 Norway; 2 Currently Statens Veteriärmedicinska Anstalt Box 7073 Uppsala S-750 07 Sweden; 3 Formerly John Curtin School of Medical Research Australian National University Canberra Australia

**Keywords:** Fetal sheep, ferritin, leucocytes, activation, MHC II, computer-assisted morphometric analysis

## Abstract

A combination of immunohistochemical techniques, a panel of monoclonal antibodies, and computer-assisted morphometric analysis was used to examine the response of the ileal Peyerr's patch of fetal lambs 7 days after treatment with ferritin *per* os. Consistent with previous studies in fetal lambs that have reported the ileal Peyer's patch to be indifferent to antigen, the present study did not find any significant changes in the size of the predominantly B-cell dome/follicle compartment or the predominantly T-cell interfollicular area, nor were differences identified in the distribution of IgM-positive (+), CD4 +, and CD8+ cells in these two compartments. However, both compartments showed a significant increase (*P* < 0.05) in the percentage of area occupied by MHC II + cells and a significant decrease (*P* < 0.05) in the percentage of area occupied by CD44+ and B5+ cells. These changes show that the ileal Peyer’s patch of fetal lambs is not indifferent to antigen and may represent the transition of a purely primary lymphoid organ to an organ that has both primary and secondary lymphoid functions.


*Developmental Immunology*, 1996, Vol 4, pp. 289–298
Reprints available directly from the publisher
Photocopying permitted by license only

© 1996 OPA (Overseas Publishers Association)
Amsterdam B.V. Published in The Netherlands
by Harwood Academic Publishers GmbH
Printed in Singapore

### Response of Leucocyte Populations in the Ileal Peyer's Patch of Fetal Lambs Treated with Ferritin *Per Os*

LENA H.M. RENSTRÖM,[†] CHARLES McL. PRESS,[‡*] WENDY TREVELLA,[§] and THOR LANDSVERK[‡]

[‡]*Department of Morphology, Genetics and Aquatic Biology, Norwegian College of Veterinary Medicine, P.O. Box 8146, Dep. 0033, Oslo, Norway.*
[†]*Currently Statens Veterinärmedicinska Anstalt, Box, 7073, S-750 07 Uppsala, Sweden.*
[§]*Formerly John Curtin School of Medical Research, Australian National University, Canberra, Australia.*

A combination of immunohistochemical techniques, a panel of monoclonal antibodies, and computer-assisted morphometric analysis was used to examine the response of the ileal Peyer's patch of fetal lambs 7 days after treatment with ferritin *per os*. Consistent with previous studies in fetal lambs that have reported the ileal Peyer's patch to be indifferent to antigen, the present study did not find any significant changes in the size of the predominantly B-cell dome/follicle compartment or the predominantly T-cell interfollicular area, nor were differences identified in the distribution of IgM-positive (+), CD4+, and CD8+ cells in these two compartments. However, both compartments showed a significant increase ($p < 0.05$) in the percentage of area occupied by MHC II+ cells and a significant decrease ($p < 0.05$) in the percentage of area occupied by CD44+ and B5+ cells. These changes show that the ileal Peyer's patch of fetal lambs is not indifferent to antigen and may represent the transition of a purely primary lymphoid organ to an organ that has both primary and secondary lymphoid functions.

KEYWORDS: Fetal sheep, ferritin, leucocytes, activation, MHC II, computer-assisted morphometric analysis.

### INTRODUCTION

In sheep, the ileal Peyer's patch (PP) produces the vast majority of circulating B cells (Gerber et al., 1986) and is responsible for the generation of the preimmune antibody repertoire (Reynaud et al., 1991). Active lymphopoiesis is present in PP from about 110 days of gestation (length of gestation in sheep is 150 days) (Reynolds and Morris, 1983) and occurs in the absence of exogenous nonself antigens. The sheep fetus is shielded from maternal immunoglobulins and even small molecules by a syndesmochorial placenta (Brambell, 1970; Boyd et al., 1976). The introduction of foreign substances into this protected environment has shown that the fetal lamb can mount an immune response to many antigens including ovalbumin, ferritin, and killed *Brucella abortus* organisms (Silverstein et al., 1963; Fahey and Morris, 1974, 1978). Although clear evidence of histological changes was present in the mesenteric lymph nodes and lamina propria of the intestine of fetal lambs following exposure to an antigen such as ferritin (Husband and McDowell, 1975; Reynolds and Morris, 1984), changes in ileal PP histology were not detected (Reynolds and Morris, 1984). The ileal PP was considered indifferent to foreign antigen, and this was argued as further support for the antigen independence of lymphopoiesis in this organ (Reynolds and Morris, 1984). Recent studies of somatic hypermutation of light-chain V genes in the ileal PP of fetuses and lambs have shown that the hypermutation process is also independent of the presence of external antigens (Reynaud et al., 1995).

However, the rate of B-cell proliferation following the introduction of foreign antigen or indeed the hypermutation process generating the repertoire does not describe the resultant antibody repertoire. Active lymphopoiesis in the ileal PP is accompanied by massive cell death and it is estimated that only 5% of PP cells produced there are destined to leave their site of production (Reynolds, 1986; Motyka and Reynolds, 1991). Thus, intense selection occurs in the PP that shapes the preimmune antibody

*Corresponding author.

### Morphometry

Computer-assisted morphometric analysis found that the mean areas of the dome/follicles and interfollicular areas of the ileal Peyer's patch of ferritin-treated fetal lambs were smaller than but not significantly different from the mean areas in the control group of fetal lambs (mean dome/follicle area [±SE] in the control group was 62,995.36 ± 28,172.38 $\mu m^2$ compared with 38,798.05 ± 8,421.32 $\mu m^2$ in the ferritin-treated group, $p > 0.05$;

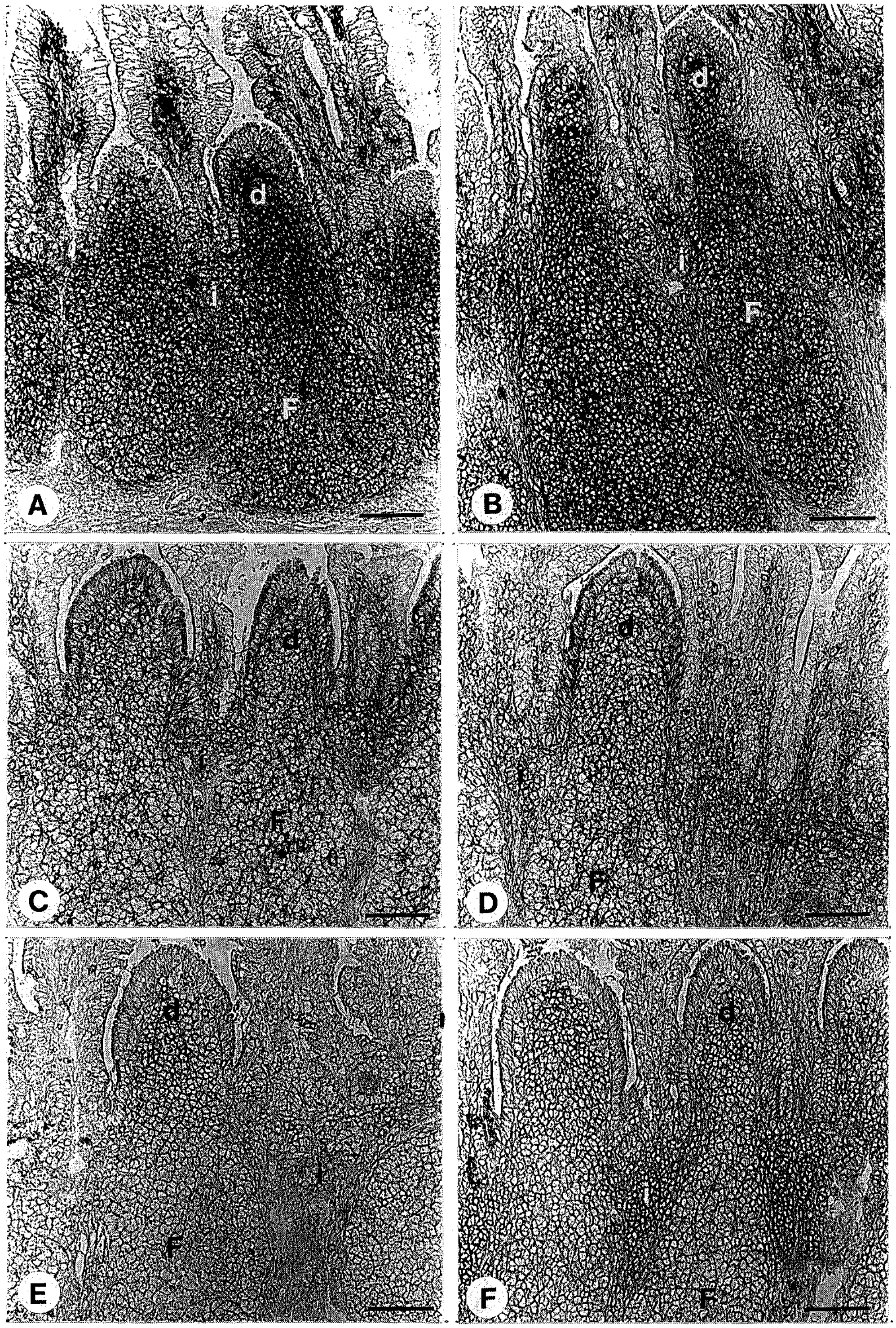

FIGURE 2. Ileal Peyer's patch. The presence of leucocyte subpopulations in fetal lambs treated with ferritin *per os* 7 days previously and in control lambs. There is a stronger presence of MHC II-positive cell populations in the dome(d) of a ferritin-treated fetal lamb (A) than in the dome of a control fetal lamb (B). A difference in the presence of CD44-positive cell populations is not obvious between a ferritin-treated fetal lamb (C) and a control fetal lamb (D). There is a weaker presence of B5-positive cell populations in the interfollicular area (i) of a ferritin-treated fetal lamb (E) than in the interfollicular area of a control fetal lamb (F). F = follicle, d = dome, i = interfollicular area. PAP immunohistochemical technique. Bar = 100 µm.

the mean interfollicular area in the control group was 9102.24 ± 1871.39 μm² compared with 5956.52 ± 608.11 μm² in the ferritin-treated group; not significant) (Fig. 3).

The mean percentages of area occupied by the leucocyte subpopulations identified by staining for IgM, CD4, and CD8 in the two compartments were also not significantly different when comparing the ferritin-treated and control groups of fetal lambs (Fig. 4). The percentage of areas occupied by T-cell subpopulations showed a tendency to increase in the dome/follicle and to decrease in the interfollicular area. The most marked decrease in T-cell subpopulations was in the percentage of area occupied by the CD4 + cell population in the interfollicular area (21.07 ± 8.27% in the control group compared with 5.70 ± 2.00% in the ferritin-treated group; not significant). Despite the widely different percentages of areas occupied by IgM + cells in the two lymphoid compartments of the ileal PP, both percentages decreased in ferritin-treated lambs (dome/follicle control 62.73 ± 7.00% compared with ferritin-treated 43.70 ± 13.63%; interfollicular area control 7.54 ± 2.92% compared with ferritin-treated 3.83 ± 1.26%; both not significant).

There was a significant increase in the mean percentage of the area staining for MHC II in both the dome/follicle (control 34.85 ± 7.14% and

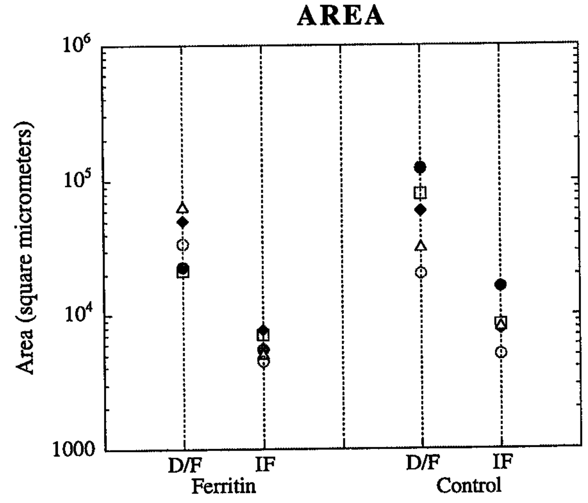

FIGURE 3. The size (μm²) of the dome/follicle (D/F) and interfollicular (IF) areas in the ileal Peyer's patch of fetal lambs treated with ferritin *per os* 7 days previously (Ferritin) and in control lambs (Control). The size of lymphoid compartments was estimated in tissue stained for 5′ nucleotidase reactivity. Within the Ferritin and Control groups in Figs. 3, 4, and 5, a symbol represents the same individual.

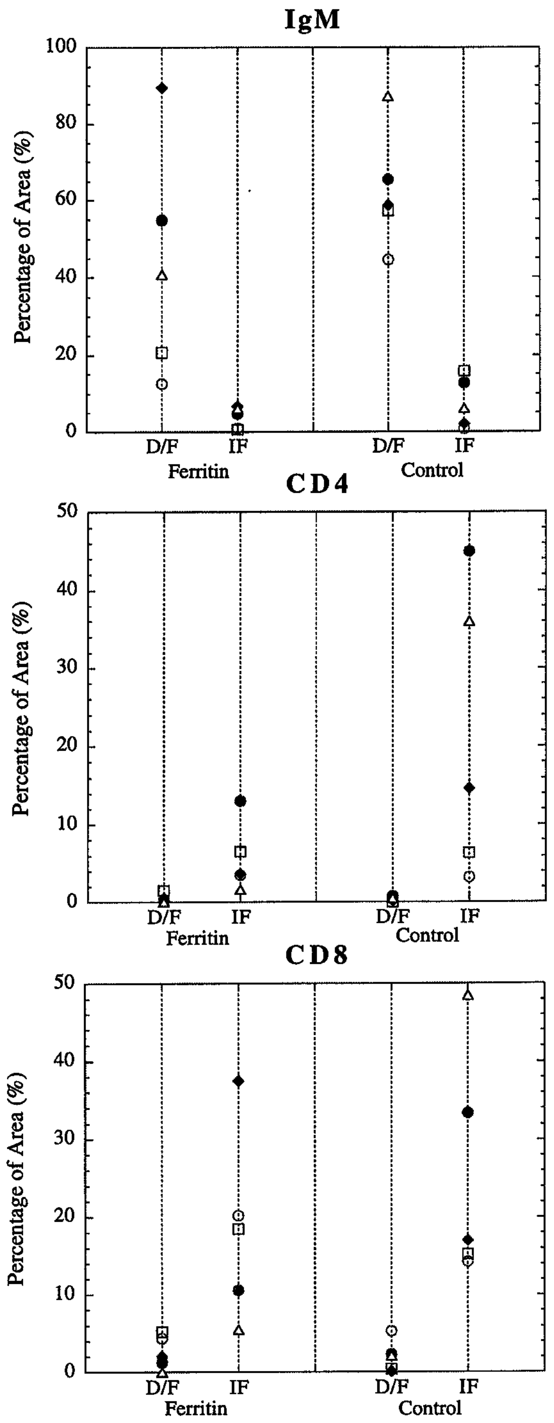

FIGURE 4. The presence (percentage of area) of leucocyte subpopulations recognized by monoclonal antibodies directed against IgM (B cells), CD4 (T helper cells), and CD8 (T cytotoxic cells) in the dome/follicle and interfollicular area of the ileal Peyer's patch of fetal lambs treated with ferritin *per os* 7 days previously (Ferritin) and in control lambs (Control). Within the Ferritin and Control groups in Figs. 3, 4, and 5, a symbol represents the same individual.

ferritin-treated 59.38 ± 7.12%; $p < 0.05$) and the interfollicular area (control 22.88 ± 4.55% and ferritin-treated 46.40 ± 4.80%; $p < 0.05$) of the ferritin-treated fetal lambs (Fig. 5). There was a tendency for a larger difference to be present between the two groups in the percentage of MHC II staining in the interfollicular area than in the dome/follicle.

The ferritin-treated group also showed a significant decrease in the mean percentage of area staining for CD44 and B5 in both compartments ($p < 0.05$; Fig. 5). The decrease in percentage of area occupied by CD44 + cells in the ferritin-treated group was greater in the dome/follicle (ferritin-treated 8.41 ± 3.80% and control 34.05 ± 6.28%) than in the interfollicular area (ferritin-treated 16.55 ± 7.78% and control 51.47 ± 10.24%). A similar tendency for the dome/follicle to show a greater change than the interfollicular area following ferritin treatment was also observed with staining for B5. The percentage of area occupied by B5 + cells in the dome/follicle of the ferritin-treated group was 7.14 ± 2.30% compared with 46.79 ± 13.39% in the control group. For the interfollicular area, the percentage of area occupied by B5 + cells in the ferritin-treated group was 16.85 ± 3.53% compared with 73.09 ± 19.00% in the control group.

### DISCUSSION

The present study shows that the ileal PP of fetal lambs is not indifferent to antigen. Following exposure to ferritin *per os*, there were significant changes in the expression of surface molecules by leucocyte populations within the lymphoid compartments of the ileal PP. Ferritin has been used in several studies in fetal lambs to induce an immune response (Silverstein et al., 1963; Husband and McDowell, 1975; Fahey and Morris, 1978; Reynolds and Morris, 1984), and although different routes of administration have been used, the oral route has been shown to induce specific antibody production (Husband and McDowell, 1975). To investigate the effect of antigen on the development of Peyer's patches, Reynolds and Morris (1984) introduced ferritin into isolated ileal segments of fetal lambs and after 7 days collected tissue samples for histological examination. These investigators did not identify changes in PP histology or size, although other lymphoid tissues such as the regional mesenteric lymph node and the lamina propria of the isolated ileum showed considerable histological changes. The findings of

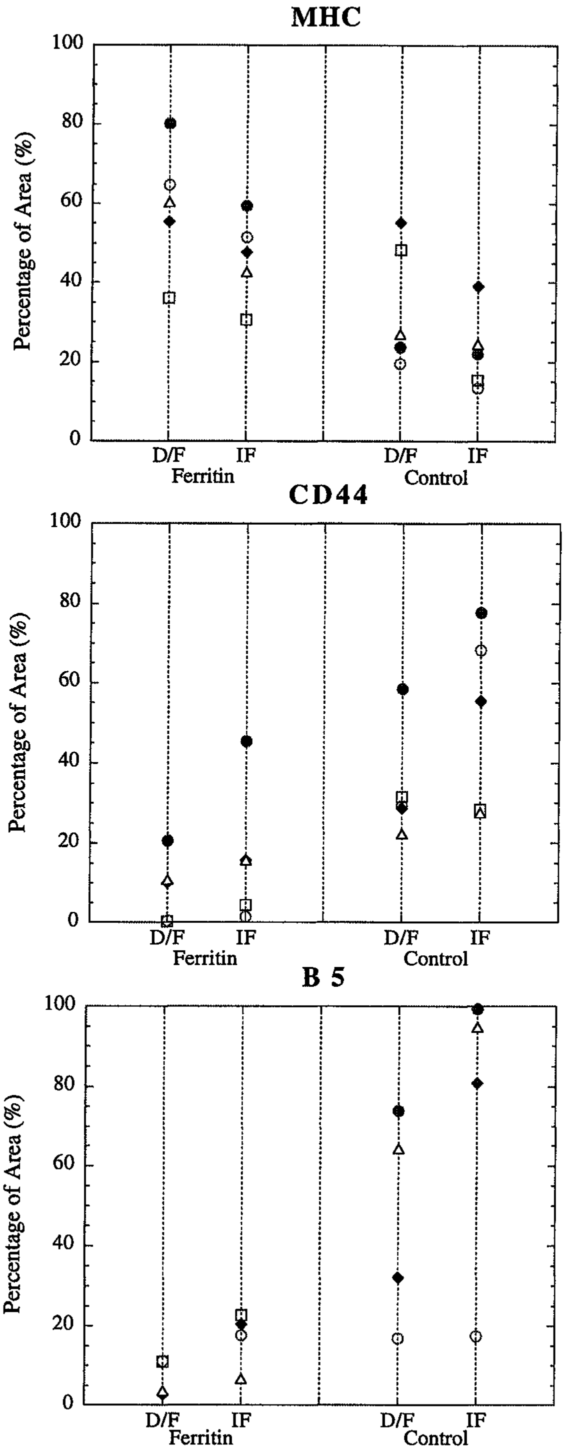

FIGURE 5. The presence (percentage of area) of leucocyte subpopulations recognized by monoclonal antibodies directed against MHC II, CD44, and B5 in the dome/follicle and interfollicular area of the ileal Peyer's patch of fetal lambs treated with ferritin *per os* 7 days previously (Ferritin) and in control lambs (Control). Within the Ferritin and Control groups in Figs. 3, 4, and 5, a symbol represents the same individual.

the present study are consistent with this previous study in that significant changes in the size of lymphoid compartments were not detected. The compartments chosen for examination in the present study were the predominantly B-cell compartment of the dome/follicle and the predominantly T-cell compartment of the interfollicular area (Larsen and Landsverk, 1986). Investigation of the percentage of area occupied by the major lymphocyte subsets, IgM + B cells and CD4 + and CD8 + T cells, in these compartments did not reveal any significant differences between the two groups of fetal lambs. However, the significance of changes that were present in these populations, such as the decreased presence of CD4 + cells in the interfollicular areas of ferritin-treated fetal lambs, may have been obscured by the large sample variation that existed within the groups. The need for fetal surgery on a large animal such as the sheep restricted the number of individuals that could be included in the present study, which may have limited the ability of the study to detect changes in these cell populations. Despite this limitation, the investigation of other leucocyte surface molecules did reveal differences between the study groups.

There was a significantly increased percentage of area occupied by MHC II + cells in the dome/follicle and interfollicular areas of the ferritin-treated fetal lambs compared with the control group. However, the increased presence of MHC II + cells in the ferritin-treated lambs, 59.4% in dome/follicle and 46.4% in interfollicular area, was still lower than the percentages reported in postnatal lambs. Hein et al. (1989) examined the surface phenotypes of lymphocytes in the ileal PP of postnatal lambs using flow cytometry and a panel of monoclonal antibodies. By using these methods, 98.3% of lymphocytes from the ileal PP were MHC II +. Increased expression of MHC II occurs with B-cell activation (Monroe and Cambier, 1983; Noelle et al., 1984; Roehm et al., 1984; Ransom and Cambier, 1986), and in the mouse, activation of protein kinase C has a role in the increased I-A antigen expression that follows B-cell activation (Monroe et al., 1984). Recent studies in sheep have shown that ileal PP B cells are rescued from apoptosis by agents that activate protein kinase C (Motyka et al., 1993). Thus, it is open to speculation whether the increased percentage of area occupied by MHC II + cells in the dome/follicle reflects a modification of the intense selection processes that occur in the follicles.

There were also significant changes in the percentage of area occupied by CD44 + and B5 + cells. The presence of both surface molecules decreased significantly in the dome/follicle and interfollicular areas of ferritin-treated fetal lambs. CD44 is a cell-adhesion molecule with proposed functions in extracellular matrix binding, cell migration, lymphopoiesis, and lymphocyte homing (Mackay et al., 1988; Lesley et al., 1993). The B5 antigen is present on the surface of T and and B lymphocytes of sheep and is involved in a pathway of T-cell activation (Hein et al., 1988). The expression of both molecules has been shown to vary between lymphocyte subpopulations and state of activation, and between lymphoid organs and the stage of ontogeny (Hein et al., 1988, 1989; Mackay et al., 1988). Thus, the decreases in expression of these molecules observed in ferritin-treated fetal lambs may be the result of the interaction of several processes. Further investigation is needed to discern the relative contributions of lymphocyte maturation, activation, and recirculation to the overall expression of these molecules within the lymphoid compartments of the ileal PP.

Nevertheless, the use of computer-assisted morphometric analysis, enabling the quantification of changes in both the interfollicular area and dome/follicle, provides some insight into the diverse processes that may be occurring in the ileal PP. With the use of flow cytometry, changes in the interfollicular area are usually obscured by the large cell populations in the follicle. The present study shows that the interfollicular area undergoes changes that differ from those occurring in the follicle. Compared with the changes in the dome/follicle, there was a larger increase in the presence of MHC II + cells and a smaller decrease in the presence of CD44 + cells and B5 + cells. A possible interpretation of these changes could be that the observed phenotypes in the interfollicular area are the result of cell activation and possibly altered levels of cell traffic whereas the observed phenotypes in the follicle are the result of changes in cell maturation. Following cell activation, the expression of MHC II, CD44 and B5 is increased (Monroe et al., 1984; Mackay et al., 1988; Hein et al., 1989) and high expression of CD44 is associated with recirculating lymphocytes, and lymphocytes that are CD44 – or $CD44^{lo}$ are those that are most sessile (Mackay et al., 1990). The reduced presence of T-cell subpopulations, especially CD4 + cells, may reflect changes in cell traffic. In postnatal lambs, the expression of B5 was low or absent in tissues containing immature B cells (Hein et al., 1989), although thymocytes from early stages of ontogeny expressed the B5 antigen (Hein et al., 1988). The percentage of area occupied by B5+ cells in the dome/follicle of ferritin-treated lambs (7.1 ± 2.3%) was closer to the percentage detected by flow cytometry in the ileal PP of postnatal lambs (10.6 ± 4.1%) (Hein et al., 1989) than the percentage of area detected in the control group of fetal lambs (46.8 ± 13.4%). A possible interpretation is that the cell populations of the dome/follicle in ferritin-treated lambs have moved toward a postnatal phenotype.

Studies in postnatal lambs have shown that the ileal PP can act as a site of contact between the immune system and antigen, leading to the production of antibody-secreting cells (Pabst and Reynolds, 1986; Reynolds, 1986). These studies in 6–9-week-old lambs concluded that PP in sheep have the characteristics of both primary and secondary lymphoid tissues. The present study, in demonstrating that the phenotype of cell populations within the fetal ileal PP is changed following exposure to antigen, focuses attention on this dual role. In an organ where the vast majority of B cells express surface Ig, the distinction between "antigen-driven" and "antigen-independent" phases in the shaping of antibody repertoires may be difficult to identify, particularly if internal self antigens participate in driving "preimmune" repertoires (Reynolds, 1987; Landsverk et al., 1990; Nicander et al., 1991). In describing the antigen independence of somatic hypermutation in the ileal PP, Reynaud et al. (1995) acknowledged the difference between the hypermutation process that generates the repertoire and the selection processes that determine the B cells that actually leave the ileal PP. Selection processes are conducted upon cell populations and occur within cellular environments (Coutinho et al., 1992). By showing changes in cell populations and cellular environments in the ileal PP, the present study may be describing the transition from a purely primary lymphoid organ to an organ that performs both primary and secondary lymphoid functions. Further studies are needed, not only to characterize the expression of other function-related leucocyte molecules in fetal lambs following exposure to exogenous antigen, but also to characterize in more detail the differences between microenvironments within the prenatal and postnatal ileal PP.

### MATERIALS AND METHODS

### Animals and Tissues

Sheep fetuses were obtained from timed matings of Norwegian Dala and Australian Merino ewes. Five of the fetuses were exposed by caesarean section under thiopentone and halothane anesthesia. A vinyl tube (external diameter 3.0 mm, internal diameter 2.0 mm) was introduced into the mouth of each fetus, passed into the esophagus and 2 ml cadmium-free ferritin (Boehringer Mannheim, 50 mg/ml) in 8 ml PBS administered. After 7 days and following euthanasia, tissue specimens were collected from the ileal PP of the fetuses at the attachment of the ileocaecal fold to the ileum. The tissues were embedded in Tissue-Tek OTC compound (4583; Miles Inc., Elkhart, IN) and frozen in monochlorodifluoromethane (Prestogas, ICI, Cheshire, UK) chilled in liquid nitrogen, wrapped in aluminium foil, and stored at −70°C. To protect the ileal mucosa during freezing, the tissue pieces were placed with the mucosa down onto pieces of liver. The gestational ages (±1 day) of the ferritin-treated fetuses ranged from 122–131 days (length of gestation in sheep is 150 days). Frozen tissue was also collected from the ileal PP of five untreated fetuses. The gestational ages of the control fetuses ranged from 125–135 days.

### Histochemistry

Frozen sections were stained for Perl's Prussian blue reaction to demonstrate ferric iron. The detection of reactivity for the enzyme 5′ nucleotidase has been described previously (Halleraker et al., 1990).

### Monoclonal Antibodies

The monoclonal antibodies used in this study, their target ligands, and primary references describing each antibody are shown in Table 1.

### Immunohistological Staining

An indirect immunoperoxidase staining technique described in detail by Press et al. (1991) was used to stain cryostat sections cut at 8 μm. This technique was used for the evaluation of lymphocyte subsets, namely, with antibodies directed against IgM, CD4, and CD8.

TABLE 1
Monoclonal Antibodies Used in This Study

| Ligand | Antibodies | Cells marked/function | Reference |
|---|---|---|---|
| IgM | McM9 | B cells | Beh, 1988 |
| CD4 | SBU-T4 | T helper cells | Maddox *et al.*, 1985 |
| CD8 | SBU-T8 | T cytotoxic cells | Maddox *et al.*, 1985 |
| MHC II | SBU-II | MHC class II antigens | Puri *et al.*, 1985 |
| B5 | B5-5 | T and B cells/T-cell activation | Hein *et al.*, 1988 |
| CD44 (Pgp-1) | 25-32 | Homing receptor | Mackay *et al.*, 1988 |

A peroxidase-anti-peroxidase (PAP) technique was used with antibodies directed against MHC II, B5, and CD44. Frozen sections were allowed to dry for at least 2 hr and then fixed for 10 min in 2% paraformaldehyde-lysine. Incubations were at room temperature and washes were for 5 min in Tris-buffered saline (0.05 M Tris/HCl, pH 7.6, 0.15 M NaCl; TBS). All dilutions of antibodies or blocking sera (normal rabbit serum) were in 1% bovine serum albumin/TBS. Following washing, endogenous peroxidase was inhibited by incubating in 0.05% phenyl hydrazine (P-6766; Sigma, St. Louis) for 40 min at 37°C. Following washing, a blocking serum was applied and the sections incubated for 20 min in a humid chamber. Subsequent incubations were in a humid chamber. The blocking serum was tapped off and the sections incubated overnight with a monoclonal antibody (Table 1) or TBS. The sections were washed, incubated with a secondary antibody directed against mouse IgG (Dako Z 259) for 30 min, and then washed and incubated with PAP complexes for 30 min. Following washing, peroxidase activity in the sections was detected by incubation for 10 min in a solution of 0.05 M Tris/HCl buffer, pH 7.6, containing 0.6 mg/ml diamino-benzidine hydrochloric acid (Sigma), 0.01% hydrogen peroxide (Merck, Darmstadt, Germany). The reaction was terminated by washing in distilled water and the sections were counterstained with 2% methyl green in distilled water for 10 min and a coverslip was applied with Aquamount (BDH Ltd., Poole, UK).

### Morphometry

The area of 5′ nucleotidase-positive dome/follicles in the ileal Peyer's patch was measured using a video-image analysis system (Zeus™, A/S Pixelwerks Ltd., Bergen, Norway) (Halleraker et al., 1994). The size of interfollicular regions was also estimated in sections stained for 5′ nucleotidase reactivity. If present in a section, at least five dome/follicles extending from the lumen to the muscular layer and at least five interfollicular regions were analyzed. The interfollicular region was defined as the region between two follicles that lay beneath a line joining the follicle crypt bottoms and above a line where the distance between the two follicles was shortest or where the follicles were first in apposition. The perimeter of a region was traced and the area calculated by the image analysis program (Fig. 1).

The percentage of area occupied by stained tissues was also estimated using the video-image analysis system (Press et al., 1992; Abrams and Springall, 1993). Appropriate gray-level thresholds were set to distinguish stained from unstained tissue within a traced region. The total stained area was determined and the percentage of the region occupied by stained tissue calculated. For each antibody, a mean percentage area was calculated for the dome/follicle and the interfollicular area for each fetus and an overall mean (± standard error) determined for the ferritin-treated and control groups. The differences between the means of the areas and the percentage areas of the two groups were assessed by a Student's $t$-test modified for use when the sample variances are not the same (Snedecor and Cochran, 1980). The chosen level of significance was $p = 0.05$.

### ACKNOWLEDGMENTS

The authors thank Elfy Gamst Bodahl and Ingjerd Andersen for skilled technical assistance. The authors also thank Dr. K. Beh, CSIRO, Australia, for providing the monoclonal antibody directed against sheep IgM.

*(Received November 29, 1994)*

*(Accepted July 18, 1995)*

### REFERENCES

Abrams D.-C., and Springall D.R. (1993). Image Analysis. *In: Methods of immunological analysis*, vol. 3, Cells and tissues, Masseyeff R.F., Albert W.H., and Staines, N.A., Eds. (Weinheim: VCH Verlagsgesellschaft) pp. 345-361.

Beh K.J. (1988). Monoclonal antibodies against sheep immunoglobulin light chain, IgM and IgA. *Vet. Immunol. Immunopathol.* **18**: 19–27.

Boyd R.D.H., Haworth C., Stacey T.E., and Ward R.H.T. (1976). Permeability of the sheep placenta to unmetabolized polar non-electrolytes. *J. Physiol.* **256**: 617–634.

Brambell F.W.R. (1970). The transmission of passive immunity from mother to young. *In: Frontiers of biology* (Amsterdam: North-Holland), pp. 201–233.

Cahill R.N.P., Heron I., Poskitt D.C., and Trnka Z. (1980). Lymphocyte recirculation in the sheep foetus. *In: Blood cells and vessel walls: Functional interactions* (Amsterdam, Oxford and New York: Excerpta Medica) pp. 145–166.

Cahill R.N.P., Poskitt D.C., Frost H., and Trnka Z. (1977). Two distinct pools of recirculating T lymphocytes: Migratory characteristics of nodal and intestinal T lymphocytes. *J. Exp. Med.* **145**: 420–428.

Coutinho A., Freitas A.A., Holmberg D., and Grandien A. (1992). Expression and selection of murine antibody repertoires. *Int. Rev. Immunol.* **8**: 173–187.

Fahey K.J., and Morris B. (1974). Lymphopoiesis and immune reactivity in the foetal sheep. *Serol. Haematol.* **7**: 548–567.

Fahey K.J., and Morris B. (1978). Humoral immune responses in foetal sheep. *Immunology* **35**: 651–661.

Freitas A.A., Viale A.-C., Sundblad A., Heusser C., and Coutinho A. (1991). Normal serum immunoglobulins participate in the selection of peripheral B-cell repertoires. *Proc. Nat. Acad. Sci. USA* **88**: 5640–5644.

Gerber H.A., Morris B., and Trevella W. (1986). The role of gut-associated lymphoid tissues in the generation of immunoglobulin-bearing lymphocytes in sheep. *Aust. J. Exp. Biol. Med. Sci.* **64**: 201–213.

Halleraker M., Landsverk T., and Nicander L. (1990). Organization of ruminant Peyer's patches as seen with enzyme histochemical markers of stromal and accessory cells. *Vet. Immunol. Immunopathol.* **26**: 93–104.

Halleraker M., Press C. McL., and Landsverk T. (1994). Development and cell phenotypes in primary follicles of foetal sheep lymph nodes. *Cell Tissue Res.* **275**: 51–62.

Hein W.R., Dudler L., and Mackay C.R. (1989). Surface expression of differentiation antigens on lymphocytes in the ileal Peyer's patches of lambs. *Immunology* **68**: 365–370.

Hein W.R., McClure S., Beya M.-F., Dudler L., and Trnka Z. (1988). A novel glycoprotein expressed on sheep T and B lymphocytes is involved in a T cell activation pathway. *J. Immunol.* **140**: 2869–2875.

Husband A.J., and McDowell G.H. (1975). Local and systemic immune responses following oral immunization of foetal lambs. *Immunology* **29**: 1019–1028.

Landsverk T., Trevella W., and Nicander L. (1990). Transfer of carbonic anhydrase-positive particles from the follicle-associated epithelium to lymphocytes of Peyer's patches in foetal sheep and lambs. *Cell Tissue Res.* **261**: 239–247.

Larsen H.J., and Landsverk T. (1986). Distribution of T and B lymphocytes in jejunal and ileocaecal Peyer's patches in lambs. *Res. Vet. Science* **40**: 105–111.

Lesley J., Hyman R., and Kincade P.W. (1993). CD44 and its interaction with extracellular matrix. *Adv. Immunol.* **54**: 271–335.

Mackay C.R., Maddox J.F., Wijffels G.L., Mackay I.R., and Walker I.D. (1988). Characterization of a 95,000 molecule on sheep leucocytes homologous to murine Pgp-1 and human CD44. *Immunology* **65**: 93–99.

Mackay C.R., Marston W.L., and Dudler L. (1990). Naive and memory T cells show distinct pathways of lymphocyte recirculation. *J. Exp. Med.* **171**: 801–817.

Maddox J.F., Mackay C.R., and Brandon M.R. (1985). Surface antigens, SBU-T4 and SBU-T8, of sheep T lymphocyte subsets defined by monoclonal antibodies. *Immunology* **55**: 739–748.

Monroe J.G., and Cambier J.C. (1983). B cell activation: III. B cell plasma membrane depolarization and hyper-Ia antigen expression induced by receptor immunoglobulin cross-linking are coupled. *J. Exp. Med.* **158**: 1589–1599.

Monroe J.G., Niedel J.E., and Cambier J.C. (1984). B cell activation: IV. Induction of cell membrane depolarization and hyper-I-A expression by phorbol diesters suggests a role for protein kinase C in murine B lymphocyte activation. *J. Immunol.* **132**: 1472–1478.

Motyka B., Griebel P.J., and Reynolds J.D. (1993). Agents that activate protein kinase C rescue sheep ileal Peyer's patch B cells from apoptosis. *Eur. J. Immunol.* **23**: 1314–1321.

Motyka B., and Reynolds J.D. (1991). Apoptosis is associated with the extensive B cell death in the sheep ileal Peyer's patch and the chicken bursa of Fabricius: A possible role in B cell selection. *Eur. J. Immunol.* **21**: 1951–1958.

Nicander L., Halleraker M., and Landsverk T. (1991) Ontogeny of reticular cells in the ileal Peyer's patch of sheep and goats. *Amer. J. Anat.* **191**: 237–249.

Noelle R., Krammer P.H., Ohara J., Uhr J.W., and Vitetta E.S. (1984). Increased expression of Ia antigens on resting B cells: An additional role for B-cell growth factor. *Proc. Nat. Acad. Sci. USA* **81**: 6149–6153.

Pabst R., and Reynolds J.D. (1986). Evidence of extensive lymphocyte death in sheep Peyer's patches II. The number and fate of newly-formed lymphocytes that emigrate from Peyer's patches. *J. Immunol.* **136**: 2011–2017.

Press C., McClure S., and Landsverk T. (1991). Computer-assisted morphometric analysis of absorptive and follicle-associated epithelia of Peyer's patches in sheep foetuses and lambs indicates the presence of distinct T- and B-cell components. *Immunology* **72**: 386–392.

Press C. McL., Halleraker M., and Landsverk T. (1992). Ontogeny of leukocyte populations in the ileal Peyer's patch of sheep. *Dev. Comp. Immunol.* **16**: 229–241.

Puri N.K., Mackay C.R., and Brandon M.R. (1985). Sheep lymphocyte antigens (OLA). II. Major histocompatibility complex class II molecules. *Immunology* **56**: 725–733.

Ransom J.T., and Cambier J.C. (1986). B cell activation: VII. Independent and synergistic effects of mobilized calcium and diacylglycerol on membrane potential and I-A expression. *J. Immunol.* **136**: 66–72.

Reynaud C.-A., Garcia C., Hein W.R., and Weill J.-C. (1995). Hypermutation generating the sheep Ig repertoire is an antigen-independent process. *Cell* **80**: 115–125.

Reynaud C.-A., Mackay C.R., M¨uller R.G., and Weill J.-C. (1991). Somatic generation of diversity in a mammalian primary lymphoid organ: The sheep ileal Peyer's patches. *Cell* **64**: 995–1005.

Reynolds J.D. (1986). Evidence of extensive lymphocyte death in sheep Peyer's patches: I. A comparison of lymphocyte production and export. *J. Immunol.* **136**: 2005–2010.

Reynolds J.D. (1987). Peyer's patches and the early development of B lymphocytes. *Curr. Top. Microbiol. Immunol.* **135**: 43–56.

Reynolds J.D., and Morris B. (1983). The evolution and involution of Peyer's patches in fetal and post-natal sheep. *Eur. J. Immunol.* **13**: 627–635.

Reynolds J.D., and Morris B. (1984). The effect of antigen on the development of Peyer's patches in sheep. *Eur. J. Immunol.* **14**: 1–6.

Roehm N.W., Leibson H.J., Zlotnik A., Kappler J., Marrack P., and Cambier J.C. (1984). Interleukin-induced increase in Ia expression by normal mouse B cells. *J. Exp. Med.* **160**: 679–694.

Silverstein A.M., Uhr J.W., Kraner K.L., and Lukes R.J. (1963). Foetal responses to antigenic stimulus. II. Antibody production by the foetal lamb. *J. Exp. Med.* **117**: 799–812.

Snedecor G.W., and Cochran W.G. (1980). *In: Statistical methods*, 7th ed. (Ames: Iowa State University Press).

Vakil M., Briles D.E., and Kearney J.F. (1991). Antigen independent selection of T15 idiotype during B cell ontogeny in mice. *Dev. Immunol.* **1**: 203–212.

Valil M., and Kearney J.F. (1991). Functional relationship between T15 and J558 idiotypes in BALB/c mice. *Dev. Immunol.* **1**: 213–224.

